# Ocular involvement in sporotrichosis: report of two cases in children^☆^^☆☆^

**DOI:** 10.1016/j.abd.2020.08.015

**Published:** 2021-03-21

**Authors:** Luciana Rodino Lemes, John Verrinder Veasey, Silvia Soutto Mayor, Carolina Contin Proença

**Affiliations:** Dermatology Clinic, Santa Casa de Misericórdia de São Paulo, São Paulo, SP, Brazil

**Keywords:** Child, Eye infections, fungal, Sporotrichosis

## Abstract

Sporotrichosis is a subcutaneous mycosis that affects humans and animals, with a typically subacute or chronic evolution, caused by *Sporothrix spp*., a dimorphic fungus. Although the cutaneous form is the most frequent presentation, the ocular involvement has been more frequently diagnosed in endemic areas, affecting mainly children and the elderly. Approximately 80% of affected patients have the lymphocutaneous form, while only 2.3% have conjunctival lesions, with 0.7% showing primary ocular involvement. We describe two cases of sporotrichosis with ocular involvement in children through inoculation by felines, with a good response to antifungal treatment.

## Case reports

**Figure 1:**
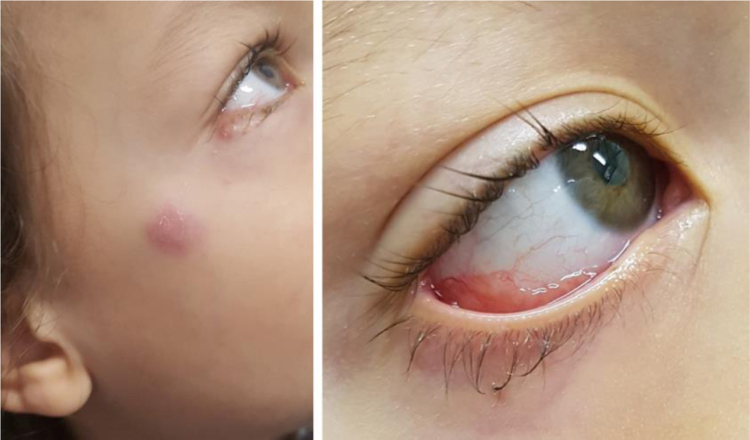
Clinical presentation of patient 1: granular erythematous lesion on the right lower tarsal conjunctiva and submandibular lymphadenopathy (Parinaud's oculoglandular syndrome).

**Figure 2:**
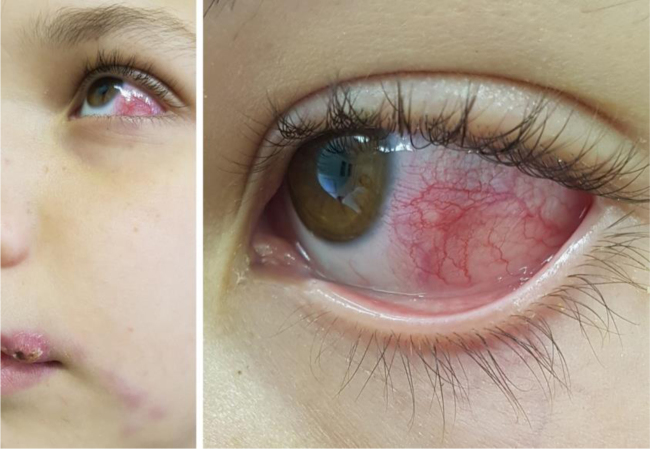
Patient 2 showing the lymphocutaneous form caused by the cat scratch on the upper lip and ocular involvement caused by sneezing of the sick cat.

**Figure 3:**
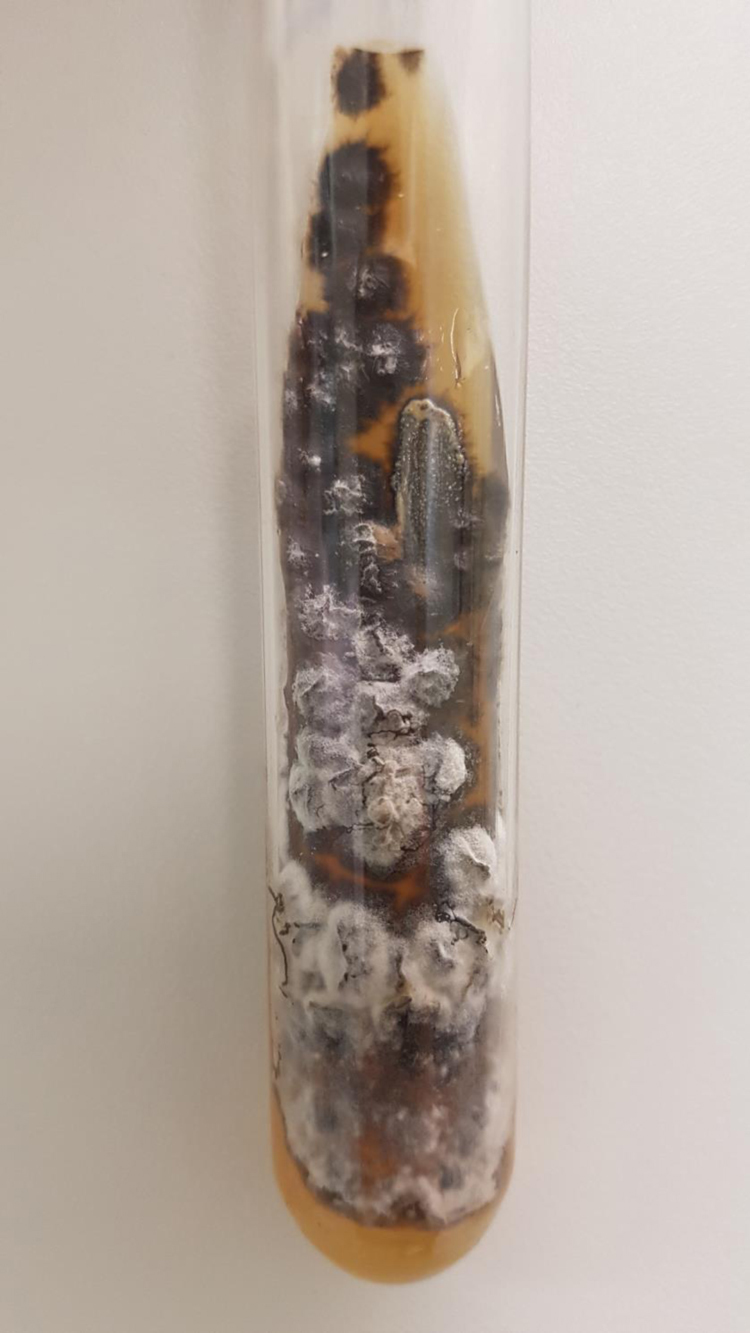
Ocular secretion culture in Sabouraud agar, showing the growth of a black and white filamentous colony.

The first case is a three-year-old male patient living with a sick cat. The patient had an eye lesion in the lower portion of the tarsal conjunctiva that progressed with lymphatic dissemination, the formation of a malar nodule and ipsilateral submandibular lymph node enlargement (Fig. 1). The second case is a 12-year-old male patient, also contaminated through contact with a sick feline, with scratches on his lip and the ocular mucosa due to probable airborne contagion through sneezing by the cat. It progressed with nodules forming a path in the lower portion of the face and conjunctivitis on the left eye (Fig. 2). In both cases, *Sporothrix spp*. was isolated from the ocular secretion by culture and showed complete resolution after treatment with itraconazole (Fig. 3).

## Discussion

Ocular involvement in sporotrichosis can occur through hematogenous dissemination, leading to intraocular injury, or by inoculation/trauma, compromising the ocular adnexae. Retrobulbar lesions seem to be more related to hematogenous dissemination, whereas anterior lesions are more associated with fungal inoculation.^1^

Both patients denied eye trauma, and the second patient categorically referred to contagion by cat sneezing on his face. Droplets from sick cats, whose secretions contain large amounts of fungi, are known to reach human membranes without the need of a local lesion for inoculation.^2^, ^3^ Interestingly, both patients were children, the age group that maintains intimate contact with domestic animals, often taking them close to the face, thus favoring fungal inoculation on the face and ocular mucosa.

The first patient had Parinaud's oculoglandular syndrome. It is a rare clinical condition, characterized by unilateral granulomatous conjunctivitis, accompanied by preauricular or submandibular satellite lymphadenopathy.^4^ In the second case, the patient had two clinical forms: the lymphocutaneous, caused by the cat scratch, and the mucosal, through spore inoculation by the droplets of the same cat on the left eye.^1^, ^2^

The approach to ocular sporotrichosis is similar to that of the cutaneous type.^1^, ^3^ The diagnosis is made by collecting conjunctival secretion using a sterile swab, followed by culture to screen for fungi.^1^, ^2^ The drugs indicated for sporotrichosis treatment are: itraconazole, potassium iodide, terbinafine, and amphotericin B. Ocular involvement in sporotrichosis should be treated with antifungals at the doses recommended for the cutaneous forms. The choice of the drug will depend on contraindications, availability and the host’s clinical conditions. Itraconazole has been the first choice at a dose of 100 to 200 mg/day until complete resolution of the lesions (or for another 2 to 4 weeks), in general, for a total of 3 to 6 months.^1^ Treatment duration varies in the literature and is determined by the patient's clinical response.

## Financial support

None declared.

## Authors’ contributions

Luciana Rodino Lemes: Drafting and writing of the manuscript; review of the manuscript.

John Verrinder Veasey: Drafting and writing of the manuscript; intellectual participation in the propaedeutics and therapeutic conduct of the cases; review of the manuscript; approval of the manuscript.

Silvia Soutto Mayor: Intellectual participation in the propaedeutics and therapeutic conduct of the cases; review of the manuscript; approval of the manuscript.

Carolina Contin Proença: Intellectual participation in the propaedeutics and therapeutic conduct of the cases; review of the manuscript.

## Conflicts of interest

None declared.
